# Plant-derived nanovesicles in kidney disease: emerging preclinical evidence and translational challenges

**DOI:** 10.3389/fphar.2026.1969534

**Published:** 2026-10-13

**Authors:** Haicheng Liu, Jingyi Zhao, Xin Cheng, Kecheng Lou, Yuwei Zhong

**Affiliations:** 1 Department of Urology, The Sixth People’s Hospital of Huizhou, Huizhou, Guangdong, China; 2 Department of Intensive Care Unit, Lanxi People’s Hospital, Jinhua, Zhejiang, China; 3 Department of Urology, The Affiliate Cancer Hospital of Gannan Medical University, Ganzhou, Jiangxi, China; 4 Department of Urology, Lanxi People’s Hospital, Jinhua, Zhejiang, China

**Keywords:** acute kidney injury, diabetic kidney disease, nephrolithiasis, plant exosome-like nanoparticles, plant-derived nanovesicles

## Abstract

Plant-derived nanovesicles (PDNVs) are lipid-bilayer nanoparticles isolated from plant extracellular fluids, juices, or tissues. Preparations obtained from disrupted tissues may contain both extracellular vesicles and membrane particles formed during processing, making product identity central to interpretation. Seven direct renal studies have examined native or engineered PDNVs in cisplatin- and sepsis-associated acute kidney injury, diabetic kidney disease, hyperuricemia-associated renal injury, and calcium oxalate nephrolithiasis. Current evidence supports three overlapping modes of use: renal cellular protection, oral modulation of intestinal and metabolic pathways, and engineered cargo delivery. Reported effects involve mitochondrial function, endoplasmic reticulum stress, ferroptosis, inflammation, microbiota, and host metabolism, but mechanistic support is uneven. SLC7A11 perturbation provides functional evidence for ferroptosis in one model, whereas most microbiota-metabolite-kidney relationships remain associative. Fluorescent tissue signals also do not establish intact-vesicle delivery or cell-specific kidney targeting. This review distinguishes direct renal PDNV evidence from adjacent plant-bioactive nanomedicines and phytochemical-modified mammalian extracellular vesicles, and critically examines product identity, preparation, dose, biodistribution, mechanism, safety, and clinical translation. No registered renal PDNV trial was identified; existing human studies concern nonrenal mucosal or intestinal applications and plant-vesicle-assisted cargo delivery. Progress toward renal translation will depend on reproducible manufacturing, mechanism-linked potency, quantitative biodistribution, causal validation, and repeated-dose safety.

## 1 Introduction

### 1.1 Clinical burden of kidney disease and unmet therapeutic needs

Acute kidney injury (AKI) and chronic kidney disease (CKD) are not isolated disease entities. AKI can reduce the glomerular filtration rate, disrupt homeostasis, and cause multiorgan complications within hours to days, whereas a single severe episode or recurrent mild episodes markedly increase the subsequent risks of CKD, kidney failure, and death (GBD Chronic Kidney Disease Collaboration, 2020; Webster et al., 2017; Jha et al., 2013; Kellum et al., 2021; Ronco et al., 2019; Khwaja, 2012; Hoste et al., 2018; Coca et al., 2012; Chawla et al., 2014; Venkatachalam et al., 2015). Cisplatin remains an important chemotherapeutic agent for many solid tumors, but its antitumor efficacy is persistently constrained by dose-limiting nephrotoxicity. Active cisplatin uptake by proximal tubular cells, mitochondrial injury, reactive oxygen species (ROS) accumulation, DNA damage, amplification of inflammation, and multiple forms of regulated cell death collectively drive renal dysfunction (Tang et al., 2023; Pabla and Dong, 2008; Miller et al., 2010). Sepsis-associated AKI commonly occurs during severe infection; its pathology cannot be reduced to hypoperfusion-induced necrosis but instead reflects the combined effects of microcirculatory dysfunction, immune dysregulation, metabolic reprogramming, mitochondrial impairment, and disrupted intercellular signaling (Peerapornratana et al., 2019; Poston and Koyner, 2019).

In chronic disease, persistent tubular injury, capillary rarefaction, inflammatory-cell infiltration, fibroblast activation, and extracellular matrix deposition converge on renal fibrosis as a common final pathway (Liu, 2011; Humphreys, 2018; Duffield, 2014). Diabetic kidney disease additionally involves hyperglycemia, advanced glycation end products, glomerular hyperfiltration, and disordered lipid metabolism (Alicic et al., 2017), whereas nephrolithiasis is sustained by reciprocal interactions among urinary supersaturation, crystal nucleation and aggregation, tubular epithelial injury, and local inflammation (Khan et al., 2016). Despite their different causes, these disorders share oxidative stress, mitochondrial damage, inflammation, cell death, and fibrosis. A natural nanosystem capable of carrying lipids, proteins, small RNAs, and plant secondary metabolites may therefore provide a complementary multicomponent strategy for complex kidney injury rather than replace established single-target therapies.

### 1.2 Why plant-derived nanovesicles are of interest as renal nanomedicines

Liposomes, poly(lactic-co-glycolic acid) (PLGA) particles, polymeric micelles, and inorganic nanomaterials can improve the solubility, stability, and tissue exposure of therapeutic cargoes, but their performance depends on material composition, manufacturing reproducibility, and *in vivo* clearance (Desai, 2012; Anselmo and Mitragotri, 2016; Mitchell et al., 2021; Park, 2013; Blanco et al., 2015). The kidney presents an additional challenge because nanoscale size alone does not confer renal targeting. Glomerular filtration, peritubular access, tubular uptake, protein corona formation, and injury-related vascular changes all influence renal exposure. For chronic kidney disease, the safety of the carrier during repeated administration is therefore as important as the activity of the payload.

Extracellular vesicles (EVs) provide a biologically derived alternative because their lipid membranes and endogenous cargoes can participate in cell-cell communication (Théry et al., 2018; Welsh et al., 2024; Lötvall et al., 2014; van Niel et al., 2018; Kalluri and LeBleu, 2020; Yáñez-Mó et al., 2015; Raposo and Stoorvogel, 2013). Plant-derived preparations are attractive because plants offer abundant source material and, for selected edible sources, compatibility with oral administration. They may also carry source-specific lipids, proteins, small RNAs, and secondary metabolites. These features are potential advantages rather than evidence of superiority over synthetic nanoparticles or mammalian EVs. Plant preparations also raise a product-definition problem: vesicles released by intact plant cells are not equivalent to membrane particles generated during grinding, pressing, or homogenization (Jeppesen et al., 2019; Kowal et al., 2016; Van Deun et al., 2017; Vestad et al., 2017; Gardiner et al., 2016; Visnovitz et al., 2019). Here, PDEV refers to preparations supported by extracellular release or other evidence consistent with EV biogenesis, whereas PDNV is used more broadly for lipid-bilayer nanoparticles isolated from plant extracellular fluids, juices, or tissues when precise biogenesis has not been established.

### 1.3 Review scope and evidence boundaries

The literature on plant-derived nanovesicles includes several biologically distinct systems that are often discussed together. Native or tissue-derived plant nanovesicles differ from synthetic nanoparticles carrying isolated phytochemicals and from mammalian extracellular vesicles obtained after phytochemical treatment of donor cells. These systems may inform drug loading, membrane engineering, or biological mechanisms, but they are not equivalent to PDNVs because their membrane origin, cargo formation, and pharmacokinetic behavior differ. Engineered PDNVs retain a plant-derived vesicular membrane while incorporating exogenous cargo or surface modification; particles reconstructed from extensively disrupted or extracted plant lipids are treated as plant-lipid nanocarriers rather than native PDNVs.

This review gives greatest weight to studies in which native or engineered PDNVs were tested directly in renal cells, kidney-disease models, or kidney-related outcomes. Extra-renal PDNV studies are used when they clarify gastrointestinal stability, cellular uptake, immune regulation, or cargo delivery, whereas synthetic plant-bioactive nanomedicines, phytochemical-modified mammalian EVs, decoction-derived particles, and fungal vesicles are used only as technical or mechanistic context. The literature search was conducted primarily in PubMed/MEDLINE and supplemented by journal websites, ClinicalTrials.gov, and reference tracking; the search was updated through 20 July 2026, and the cited clinical-trial records were rechecked on 6 August 2026. Because the renal studies differ substantially in plant source, purification, dose expression, administration route, biodistribution methods, and endpoints, the evidence was synthesized qualitatively. The current literature falls broadly into three overlapping therapeutic directions: local renal protection, oral modulation of the gut-kidney axis, and engineered delivery using plant-derived vesicular membranes. These directions organize the evidence without implying distinct biological classes (Figure 1).

**FIGURE 1:**
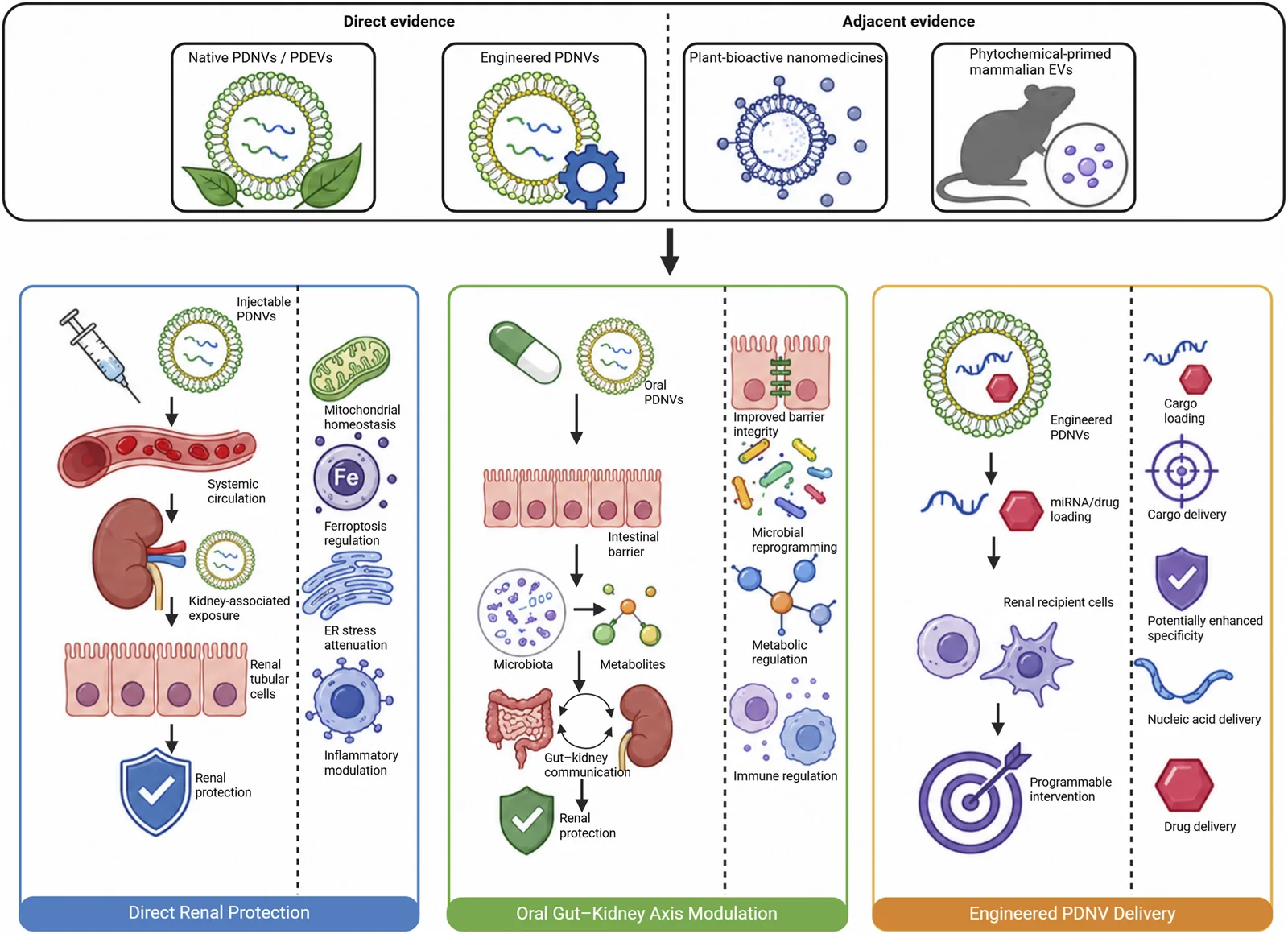
Evidence boundaries and overlapping therapeutic directions of plant-derived nanovesicles in kidney disease. The upper distinguishes direct evidence derived from native or engineered PDNVs from adjacent evidence derived from plant-bioactive nanomedicines and phytochemical-modified mammalian extracellular vesicles. The lower summarizes three overlapping modes of use in the renal literature: renal cellular protection, oral gut-kidney modulation, and engineered cargo delivery. Kidney-associated signals indicate tissue exposure and should not be interpreted as proof of intact-vesicle targeting. PDNV, plant-derived nanovesicle; PDEV, plant-derived extracellular vesicle; EV, extracellular vesicle.

## 2 Sources, preparation, and fundamental characteristics of plant-derived nanovesicles

### 2.1 Plant sources and nomenclature

PDNVs have been obtained from fruits, vegetables, and medicinal plants. Frequently studied edible sources include grape, grapefruit, lemon, orange, blueberry, tea, ginger, garlic, carrot, tomato, strawberry, and bitter melon, whereas medicinal sources include ginseng, honeysuckle, dandelion, *Atractylodes macrocephala*, *Salvia miltiorrhiza*, and other medicinal herbs (Pinedo et al., 2021; Fan et al., 2022; Dad et al., 2021; Kim et al., 2022; Cao et al., 2023; Sarasati et al., 2023; Mu et al., 2023; Calzoni et al., 2024; Rodríguez de Lope et al., 2025; Thieron et al., 2024; Shokooh and Fereidoni, 2025; Viršilė et al., 2022; Xiao et al., 2018). Edible fungi can also produce nanovesicles with similar morphology and delivery properties, but fungi are not taxonomically plants and should be discussed separately as other food-derived extracellular vesicles rather than included merely to expand the PDNV evidence base. Particles from different sources differ substantially in lipid composition, proteome, small-RNA profile, and secondary metabolites, which in turn influence gastrointestinal stability, cellular uptake, and immunomodulation. Ginger vesicles are enriched in phosphatidic acid, gingerols, and related lipids and often exhibit colon tropism and anti-inflammatory activity. Citrus vesicles contain abundant phospholipids and plant small RNAs. Vesicles from medicinal plants may also carry saponins, flavonoids, phenolic acids, or terpenoids, but such cargoes must be demonstrated by mass spectrometry or quantitative assays rather than inferred from the pharmacology of the source plant.

The terms exosome, exosome-like nanoparticle, extracellular vesicle-like nanoparticle, and nanovesicle are used in parallel across the field. Under the MISEV framework, the term exosome, which implies a specific biogenesis pathway, should be used only when an endosomal multivesicular-body origin has been demonstrated (Théry et al., 2018; Welsh et al., 2024; Lötvall et al., 2014). For preparations obtained from plant juice or tissue homogenate without biogenetic validation, plant-derived extracellular vesicle-like nanoparticles or plant-derived nanovesicles are more defensible terms. This is not merely a semantic distinction; it underpins reproducibility and attribution of biological activity. If a preparation contains fragments of chloroplasts, mitochondria, endoplasmic reticulum, or plasma membrane, the observed activity may derive from a heterogeneous particle mixture rather than a defined EV subpopulation (Pinedo et al., 2021).

### 2.2 Isolation and purification

Most studies begin with washing fresh tissue, mechanical homogenization or pressing, and low-speed centrifugation to remove coarse debris. Centrifugation at 10,000–20,000 × g is then used to remove organelles and larger particles, followed by ultracentrifugation at 100,000–150,000 × g to pellet nanovesicles. This workflow is accessible and straightforward, but the pellet can simultaneously enrich protein aggregates, polysaccharides, pigments, and organelle membrane fragments. Density-gradient centrifugation provides further separation by buoyant density, size-exclusion chromatography gently removes soluble proteins, and tangential-flow filtration is more suitable for scale-up. Although polymer precipitation is convenient, residual polymer may affect downstream functional assays (Fan et al., 2022; Thieron et al., 2024; Shokooh and Fereidoni, 2025; Viršilė et al., 2022; Xiao et al., 2018). Pretreatment conditions have an especially large effect on yield and purity for plant materials rich in pectin, cellulose, or starch. He et al. systematically reviewed extraction methods for plant-derived vesicle-like nanoparticles (PDVLNs) and their application across diseases (He et al., 2023). Their comparison of ultracentrifugation and density-gradient centrifugation and discussion of source-specific pretreatment strategies provide a useful methodological reference for renal studies.

PDEVs collected from culture supernatants or extracellular fluids of intact plant cells are not equivalent to PDNVs obtained after high-energy disruption of whole tissues. The former more closely represent actively released EVs, whereas the latter are more likely to contain artificially formed membrane vesicles. Nanoparticles detected in traditional herbal decoctions raise an additional conceptual issue: heating can disrupt native lipid bilayers and allow proteins, polysaccharides, saponins, and small molecules to reassemble during cooling. Nanoparticles from compound decoctions should therefore be discussed separately as self-assembled natural nanostructures rather than labeled directly as plant exosomes.

### 2.3 Characterization and quality control

Basic PDNV characterization should cover morphology, size and concentration, surface potential, membrane architecture, and major components. Transmission electron microscopy or cryo-electron microscopy can visualize cup-shaped or near-spherical structures. Nanoparticle tracking analysis (NTA) and dynamic light scattering (DLS) are used to measure size, but they rely on different physical principles and their results are not interchangeable. Zeta potential reflects colloidal stability but cannot substitute for purity assessment. Many studies report particles concentrated within 30–200 nm, although plant-derived preparations may have broader distributions that are strongly affected by centrifugation and filtration conditions (Gardiner et al., 2016; Fan et al., 2022; Xiao et al., 2018).

CD9, CD63, CD81, TSG101, and ALIX are commonly used as supporting markers for mammalian EVs, but they are not universal markers of plant vesicles. HSP70, annexin-like proteins, PATELLIN, TET8, and PEN1 have been reported in selected plant EV subpopulations, yet their cross-species conservation and antibody compatibility are limited (Pinedo et al., 2021; Thieron et al., 2024). More informative quality control should therefore report particle number, total protein, total lipid, mass of source tissue, final yield, and ratios such as particles-to-protein and particles-to-lipid. Functional experiments should also include matched free extract, membrane disruption, heat inactivation, RNase or protease treatment with membrane permeabilization, and synthetic liposome controls to determine whether activity depends on intact vesicles, surface molecules, or free constituents.

### 2.4 Stability, oral absorption, and biodistribution

Oral compatibility is a frequently proposed advantage of selected PDNV preparations, although gastrointestinal stability and systemic exposure are strongly source- and formulation-dependent. Particles derived from grape, ginger, and grapefruit show varying resistance to acid and enzymes, mucus penetration, and uptake by intestinal epithelial cells or macrophages in gastrointestinal models (Ju et al., 2013; Wang et al., 2014; Zhuang et al., 2015; Zhang et al., 2016a; Teng et al., 2018; Chen et al., 2019; Raimondo et al., 2015). Their phospholipid composition, surface glycans, and proteins may participate in cellular recognition, and some particles may be utilized by the gut microbiota or alter microbial metabolism. At least three routes of action are plausible: local anti-inflammatory activity in the intestine; trans-epithelial entry of vesicles or their cargoes into the circulation; and indirect renal effects through microbiota and metabolite remodeling along the gut–kidney axis. Drawing on the systematic analysis of oral EV delivery by Song et al. (2022), gastrointestinal stability, mucus penetration, epithelial transport, and local immune effects should be evaluated separately. PDNV absorption should not be inferred solely from fluorescence in feces or blood; each step requires dedicated validation.

Stability in the gastrointestinal tract does not imply that large quantities of intact vesicles enter the systemic circulation. Whether dietary plant miRNAs reach mammalian tissues across species at physiologically relevant concentrations remains controversial (Snow et al., 2013; Dickinson et al., 2013; Man et al., 2020). Fluorescent tracing may also be confounded by free dye, lipid exchange, or particle aggregation. Future studies should combine radiolabeling, stable isotopes, membrane-protein-specific tags, digital PCR, and tissue mass spectrometry, while distinguishing intact vesicles from degradation products and repackaged cargoes. For renal applications, it is also necessary to determine whether particles enter the urinary space by glomerular filtration, are taken up from the peritubular capillary side, or exert indirect effects after processing by the hepatic and splenic reticuloendothelial system.

### 2.5 Vesicle cargo composition and source specificity

PDNVs are not merely empty lipid spheres. Their membrane lipids govern particle stability, gastrointestinal tolerance, and cellular uptake, whereas proteins, mRNAs, small RNAs, DNA fragments, and plant secondary metabolites may be carried within the lumen or on the membrane surface (Fan et al., 2022; Dad et al., 2021; Kim et al., 2022; Cao et al., 2023; Sarasati et al., 2023; Mu et al., 2023; Calzoni et al., 2024; Rodríguez de Lope et al., 2025). Lipid composition differs especially markedly among plant sources. Variable proportions of phosphatidic acid, phosphatidylcholine, phosphatidylethanolamine, and galactolipids affect curvature, fusion, and receptor recognition. Certain subpopulations of ginger-derived particles show strong intestinal mucosal uptake and anti-inflammatory effects; citrus particles are relatively stable under acidic conditions; and tea-derived particles are rich in polyphenols and antioxidant activity. Even within the same species, particles released from roots, stems, leaves, fruits, or cultured cells may have distinct multi-omic profiles. A plant name cannot substitute for a product definition.

Small RNAs are among the most intensively studied and most frequently overstated PDNV cargoes. An early report proposed that dietary plant MIR168a could regulate mammalian LDLRAP1 across kingdoms, generating an influential hypothesis of cross-kingdom RNA communication (Zhang et al., 2012). Several subsequent studies, however, failed to reproduce stable and sufficient systemic exposure under ordinary feeding conditions (Snow et al., 2013; Dickinson et al., 2013). The ability of a plant miRNA to regulate gene expression in mammalian cells *in vitro* therefore does not establish intact absorption and systemic activity at dietary doses. Studies of cross-kingdom RNA regulation should answer four questions: Is the target RNA truly enclosed within the vesicle? Can it be detected in recipient cells as an absolute copy number after administration? Is the achieved concentration sufficient for a biological effect? Does knockdown or removal of the RNA reduce vesicle efficacy? Sequence prediction and changes in target-gene expression alone cannot establish that an RNA is the dominant effector.

Plant secondary metabolites may be a key distinction between PDNVs and mammalian EVs. Gingerols, naringin, polyphenols, saponins, and phenolic acids can insert into membranes or be encapsulated within particles, potentially improving the stability and delivery of poorly soluble compounds. However, free small molecules often co-sediment with vesicles. Without size-exclusion or density-based purification, co-precipitated plant extract may be misidentified as vesicular cargo. Dialysis, ultrafiltration, or chromatography should be used to remove free compounds, and encapsulation should be confirmed by quantification before and after membrane disruption, analysis of intravesicular *versus* extravesicular distribution, and release profiling.

### 2.6 Storage, reconstitution, and transport

Advancing PDNVs from laboratory preparation to drug development requires robust storage stability. Short-term storage at 4 °C may promote lipid oxidation, protein degradation, and particle aggregation, whereas repeated freeze-thaw cycles can disrupt membranes and alter particle size. Storage at −80 °C is common but does not automatically preserve long-term potency. Because vesicles from different sources vary in their tolerance to temperature and osmotic stress, shelf life should be defined by particle size, particle recovery, membrane integrity, key cargoes, and *in vitro* potency rather than appearance alone.

Lyophilization is a promising formulation strategy. Cryoprotectants such as sucrose and trehalose can reduce membrane fusion and aggregation after reconstitution, although protectant ratio, freezing rate, and residual moisture all affect product quality. For oral products, lyophilized powder facilitates ambient transport and quantitative filling. Injectable products must additionally meet requirements for sterility, visible and subvisible particulates, and osmolality. Investigators should report the number of freeze-thaw cycles and storage duration and should not combine freshly prepared and long-stored samples in the same experiment. These differences in source, processing, storage, and product definition are consolidated in the conceptual matrix in Table 1 which provides the foundation for the evidence analysis that follows.

**TABLE 1:** Conceptual boundaries in plant-derived nanovesicle research.

Category	Typical source or preparation	Membrane/cargo origin	Evidence class	Use in this review
Native PDEV/PDNV	Plant extracellular fluid, juice, or tissue isolate	Naturally released vesicles and/or tissue-derived membrane particles	Direct evidence when isolation and product identity are described	Renal efficacy and mechanism
Engineered PDNV	Native PDNV loaded with miRNA, siRNA, or drug	Plant-membrane chassis plus exogenous cargo	Direct evidence, with engineering evaluated separately	Cargo loading, release, delivery, and safety
Plant-constituent nanomedicine	Curcumin nanoparticles, honokiol liposomes, or ganoderic-acid PLGA	Synthetic carrier plus plant small molecule	Adjacent evidence	Pharmacological and technical support
Phytochemical-primed mammalian EV	MSC-EVs collected after curcumin or resveratrol treatment	Mammalian membrane and endogenous cargo	Adjacent evidence	Mechanistic extrapolation
Decoction-derived self-assembled particle	Composite particles formed after high-temperature decoction	Self-assembled polysaccharides, proteins, saponins, and small molecules	Generally outside PDNV scope	Separate traditional-formulation nanostructure
Fungal or other food-derived vesicle	Truffle, shiitake, and other fungal materials	Fungal membrane and native cargo	Outside PDNV scope	Comparative food-vesicle evidence

Abbreviations: EV, extracellular vesicle; MSC, mesenchymal stromal cell; PDNV, plant-derived nanovesicle; PDEV, plant-derived extracellular vesicle; PLGA, poly(lactic-co-glycolic acid).

## 3 Direct preclinical evidence for plant-derived nanovesicles in kidney disease

### 3.1 Honeysuckle-derived exosome-like nanovesicles in cisplatin-induced acute kidney injury

Gong et al. evaluated honeysuckle-derived exosome-like nanovesicles (HELNVs) in cisplatin-treated HK-2 cells and mice with cisplatin-induced AKI (Gong et al., 2026). HELNVs reduced ROS accumulation, inflammatory cytokines, and apoptosis *in vitro* and lowered serum creatinine and blood urea nitrogen while improving renal histology and mitochondrial readouts *in vivo*. A kidney-associated tracer signal peaked at approximately 8–10 h after intraperitoneal administration. The study therefore links renal functional improvement with mitochondrial and inflammatory changes, although the tracer experiment does not establish intact-vesicle delivery to renal cells.

The active vesicular component remains undefined, and the contribution of intact HELNVs has not been separated from co-isolated plant constituents. Comparisons with vesicle-depleted honeysuckle preparations and matched lipid particles would clarify this point. In cisplatin models, renal protection also needs to be interpreted alongside antitumor activity because suppression of oxidative stress or apoptosis in the kidney should not compromise the intended anticancer effect.

### 3.2 Lemon-derived extracellular vesicle-like nanoparticles in nephrolithiasis

Zhang et al. reported that lemon-derived extracellular vesicle-like nanoparticles (LEVNs) attenuated calcium oxalate nephrolithiasis (Zhang et al., 2023). In crystal-stimulated tubular epithelial cells and animal models, LEVNs reduced endoplasmic reticulum stress, cellular injury, crystal adhesion, and renal crystal deposition. These findings extend the biological effects of lemon-derived preparations beyond changes in urinary chemistry and identify tubular stress responses as a possible vesicle-sensitive process.

The vesicle effect, however, is difficult to separate from the established citrate-related effects of lemon preparations. Clinical studies of lemon juice have focused on urinary citrate and stone recurrence rather than vesicle exposure (Ruggenenti et al., 2022; Aras et al., 2008). Purified LEVNs therefore require direct comparison with citrate-matched solutions, whole lemon preparations, and vesicle-depleted supernatants before the vesicular component can be assigned an independent anti-stone effect.

### 3.3 Dandelion-derived exosome-like nanovesicles in sepsis-associated AKI

Dandelion-derived exosome-like nanovesicles reduced renal histological injury, lipid peroxidation, and inflammatory readouts in a sepsis-associated AKI model while increasing SLC7A11 expression (Zhang et al., 2026). Interference with SLC7A11 weakened the protective effect, providing functional evidence that ferroptosis regulation contributes to the response rather than merely accompanying it.

This is currently one of the clearer pathway-perturbation results in the renal PDNV literature. It does not identify the vesicular cargo responsible for SLC7A11 regulation, and the relevance of the effect across renal cell populations remains uncertain. In sepsis, any anti-inflammatory or antiferroptotic intervention also has to be evaluated in the context of pathogen control and standard supportive treatment.

### 3.4 Engineered *Atractylodes macrocephala* vesicles for miR-146a-5p delivery

Yan et al. loaded miR-146a-5p into *A. macrocephala*-derived vesicles by electroporation to generate AMEVLP@miR for sepsis-associated AKI (Yan et al., 2026). Oral treatment improved renal function and histology and reduced inflammatory, oxidative-stress, mitochondrial, and apoptotic injury. The loaded preparation produced greater improvement than unloaded vesicles in several readouts. Kidney-associated fluorescence, including colocalization with AQP1-positive tubular structures, was also reported, together with changes in the gut microbiota.

AMEVLP@miR is better interpreted as an engineered delivery system than as a native PDNV preparation. The comparison with unloaded vesicles supports an incremental effect of engineering, but fluorescence alone does not establish that intact vesicles delivered miR-146a-5p to proximal tubular cells. Encapsulation, release, and recipient-cell delivery of the introduced RNA remain central to the mechanistic interpretation, particularly because intestinal and renal effects may occur in parallel.

### 3.5 *Salvia miltiorrhiza*-derived extracellular vesicles in diabetic kidney disease

Liu et al. examined orally administered *S. miltiorrhiza*-derived extracellular vesicles (SMEVs) in mice with diabetic kidney disease (Liu et al., 2026). Differential centrifugation followed by a sucrose density gradient was used for preparation, and renal outcomes were assessed together with 16S sequencing and untargeted metabolomics. SMEVs reduced proteinuria and renal tissue injury and were accompanied by lower inflammatory and fibrotic readouts as well as changes in microbial composition and host metabolites.

The integrated design links renal improvement with intestinal and metabolic changes, but it does not establish whether either is required for the renal effect. Microbiota depletion or transplantation and replacement of candidate metabolites would be needed to distinguish causal mediators from secondary changes that follow improvement in diabetes or kidney injury.

### 3.6 *Epimedium*-derived exosome-like nanovesicles in sepsis-associated AKI

*Epimedium*-derived exosome-like nanovesicles (EENVs) attenuated sepsis-associated AKI while improving intestinal-barrier indices and altering microbial, short-chain fatty acids, lipid-metabolic, and inflammatory readouts (Yang L. et al., 2026). Kidney-associated fluorescence was also reported. The results are consistent with coordinated intestinal, metabolic, and renal responses, but the available data do not establish a linear sequence from microbiota change to renal protection.

The relative contributions of local intestinal activity, circulating metabolites, and kidney-associated vesicle or cargo exposure therefore remain unresolved. Perturbation of the microbiota or candidate metabolites would be more informative than additional parallel omics associations, while orthogonal tracking is needed before kidney-associated fluorescence is interpreted as intact-vesicle delivery.

### 3.7 *Perilla frutescens*-derived nanovesicles in hyperuricemia-associated kidney injury

*Perilla frutescens*-derived nanovesicles (PLENs) were evaluated after oral administration in hyperuricemia-associated kidney injury (Yang Z. et al., 2026). Treatment improved renal pathological and functional readouts and was accompanied by changes in intestinal barrier function, microbial and metabolite profiles, inflammatory signaling, and urate transporters including ABCG2, URAT1, and GLUT9.

This model is particularly useful for separating intestinal and renal contributions because urate balance depends on both compartments. The present data do not distinguish whether renal protection mainly reflects lower systemic urate, altered intestinal urate disposal, direct renal effects, or a combination of these processes. Selective transporter or metabolite perturbation would help resolve that distinction.

### 3.8 Comparative appraisal of the current renal evidence

The seven direct renal studies cover cisplatin- and sepsis-associated AKI, nephrolithiasis, diabetic kidney disease, and hyperuricemia-associated renal injury. Most report improvement in renal injury, but the evidence supporting the proposed mechanism differs substantially among preparations. This distinction is more informative than comparing the number of altered biomarkers across studies.

HELNVs combine functional, inflammatory, mitochondrial, and kidney-associated tracer data, but the active cargo and integrity of the renal signal remain unresolved. LEVNs link tubular uptake and endoplasmic reticulum stress with reduced crystal injury, although the vesicle contribution has not been cleanly separated from citrate and other soluble lemon constituents. Dandelion vesicles provide stronger pathway-level support because SLC7A11 interference weakens protection, placing ferroptosis closer to a functional mechanism than a correlated readout.

The oral SMEV, EENV, and PLEN studies connect renal outcomes with intestinal barrier function, microbiota, and host metabolism (Liu et al., 2026; Yang L. et al., 2026; Yang Z. et al., 2026). These datasets support gut-kidney involvement but remain largely associative. AMEVLP@miR addresses a different question: whether a plant-derived vesicular membrane can serve as a carrier for an introduced RNA (Yan et al., 2026). Its greater activity than unloaded vesicles supports an engineering contribution, although the site and form of cargo delivery remain to be resolved.

Across these studies, renal cellular protection, intestinal and metabolic modulation, and engineered cargo delivery recur as overlapping therapeutic directions. None of the current evidence supports ranking plant sources or assuming a shared renal mechanism. Differences in isolation, characterization, dose units, controls, biodistribution methods, and observation periods remain substantial and are central to the interpretation of efficacy. The principal features and evaluation priorities of these three overlapping therapeutic directions are summarized in Table 2.

**TABLE 2:** Comparison of three overlapping therapeutic directions for plant-derived nanovesicles in kidney disease.

Therapeutic direction	Representative studies	Principal development context	Demonstrated route(s)	Critical quality attributes	Core evaluation endpoints
Renal cellular protection	Honeysuckle, dandelion, and lemon systems	Acute drug-induced or inflammatory injury; calcium oxalate nephrolithiasis	Intraperitoneal, intravenous, and oral administration in current studies	Product identity; membrane integrity; particle- and mass-based dose; mechanism-linked potency	Quantitative renal exposure; relevant renal cell uptake; SCr/BUN or stone burden; mitochondrial, ferroptosis, ER-stress, and inflammatory readouts
Oral gut-kidney modulation	Salvia miltiorrhiza, Epimedium, and Perilla frutescens	Diabetic kidney disease, hyperuricemia-associated injury, and systemic inflammatory/metabolic injury	Oral administration in representative studies	Gastrointestinal stability; dose recovery; barrier- and microbiota-related potency; relevant metabolites	Barrier integrity; microbiota and metabolite changes; renal outcomes; causal testing by depletion, transplantation, metabolite replacement, or transporter perturbation
Engineered cargo delivery	Atractylodes AMEVLP@miR	Kidney injury requiring programmable nucleic-acid delivery	Oral gavage in the current proof-of-concept study	Loading/encapsulation; membrane integrity; gastrointestinal stability; release profile; free-cargo removal	Cargo-specific activity; orthogonal evidence of recipient-cell delivery; off-target effects; repeated-dose safety

The three directions overlap and are defined primarily by intended site of action and product function rather than administration route. Abbreviations: BUN, blood urea nitrogen; ER, endoplasmic reticulum; SCr, serum creatinine.

Direct renal studies now span several disease models, but cross-study comparison remains limited by heterogeneity in plant source, plant part, purification, particle characterization, dose expression, administration route, and follow-up. Reporting particle number together with mass-based measures, source-material input, batch yield, membrane integrity, and disease-specific endpoints would make future comparisons more interpretable. A comparative appraisal of the direct renal PDNV studies is provided in Table 3.

**TABLE 3:** Comparative appraisal of direct preclinical studies of plant-derived nanovesicles in kidney disease.

PDNV	Renal model	Preparation	Dose and administration	Key controls	Biodistribution	Mechanistic validation	Design	Main contribution	Main limitation
HELNVs (Gong et al., 2026)	HK-2 cells; cisplatin AKI in male C57BL/6 mice	Tissue-derived HELNVs; detailed purification and particle size not provided in the review	50–200 μg/mL *in vitro*; 30 mg/kg i.p. daily for 5 days	Control and cisplatin-injured groups; matched vesicle-depleted plant control not reported	Small-animal tracer imaging; kidney-associated signal peaked at ∼8–10 h	Mitochondrial and inflammatory improvement; no cargo-specific loss-of-function	Computer randomization; n = 6/group reported; blinding not stated	Links renal functional recovery with mitochondrial and inflammatory changes	Tracer signal does not prove intact-vesicle delivery; active cargo undefined
LEVNs (Zhang et al., 2023)	Tubular epithelial cells; calcium oxalate stone models	Juice-derived extracellular vesicle-like nanoparticles; detailed purification/size not provided in the review	Oral treatment; exact dose not reported in the review	Stone-model controls; citrate-matched and vesicle-depleted controls not reported	Tubular uptake/kidney trafficking reported	ER-stress attenuation with reduced crystal adhesion and deposition; no loss-of-function validation	Sample size, randomization, and blinding not stated in the review	Connects lemon-derived vesicles with tubular ER stress and stone progression	Independent vesicle effect not separated from citrate or soluble lemon components
Dandelion PGY-Exo (Zhang et al., 2026)	LPS-treated HUVECs; CLP sepsis-associated AKI in mice	Dandelion-derived exosome-like nanovesicles; detailed particle size not reported in the review	2 mg/kg i.v. at 4 h after CLP (verified from original report)	PBS/sepsis controls; vesicle-depleted plant control not reported	No orthogonal intact-vesicle renal localization established	SLC7A11 downregulation weakens protection, supporting ferroptosis involvement	Sample size, randomization, and blinding not stated in the review	Strongest current pathway-perturbation evidence in renal PDNV studies	Responsible vesicular cargo and renal cell specificity remain undefined
AMEVLP@miR (Yan et al., 2026)	LPS-treated HK-2 cells; sepsis-associated AKI in C57BL/6J mice	∼90-nm Atractylodes vesicles electroporated with miR-146a-5p; stable in simulated GI fluids	150 μg/mL *in vitro*; 100 mg/kg oral gavage daily for 7 days starting 24 h after LPS	Control, AKI, unloaded AMEVLP, and AMEVLP@miR groups	ICG/IVIS imaging with kidney signal and AQP1 colocalization	Loaded vesicles outperform unloaded vesicles in several readouts; cargo delivery itself not directly demonstrated	Random allocation; n = 5/group reported; blinding not stated	Direct renal proof of concept for engineered plant-vesicle cargo loading	Fluorescence does not establish intact-vesicle or miRNA delivery; gut and renal contributions unresolved
SMEVs (Liu et al., 2026)	Diabetic kidney disease in BKS.DB mice	Differential centrifugation plus sucrose density-gradient ultracentrifugation; mean diameter ∼163.6 nm	Oral administration for 6 weeks; exact dose not reported in the review	Disease control and treatment comparison; matched vesicle-depleted plant control not reported	Reported exposure primarily assessed in the gastrointestinal/oral context; renal intact-vesicle delivery not established	16S sequencing and untargeted metabolomics identify microbiota-metabolic associations	Sample size, randomization, and blinding not stated in the review	Integrates renal outcomes with microbiome and metabolome profiling	Association does not establish microbiota or metabolite mediation
EENVs (Yang et al., 2026a)	Murine sepsis-associated AKI	Epimedium-derived exosome-like nanovesicles; purification and exact dose not detailed in the review	Route/dose not detailed in the review	Disease and treatment controls; depletion/transplantation controls not reported	Kidney-associated fluorescence reported	Barrier, SCFA, LPS-translocation, lipid-metabolic, and TLR4/NF-kB changes measured in parallel	Kidney-signal comparison n = 3 in supporting data; overall sample size/randomization/blinding not stated in the review	Connects intestinal, metabolic, and renal responses in sepsis AKI	Causal sequence along the gut-kidney axis not established
PLENs (Yang et al., 2026b)	Potassium oxonate/hypoxanthine-induced hyperuricemic mice	Gastrointestinally stable Perilla-derived nanovesicles; detailed purification/size not reported in the review	Oral chronic intervention; exact dose not reported in the review	Disease and treatment controls; selective transporter perturbation not reported	Gastrointestinal-dominant exposure reported; direct renal intact-vesicle delivery not established	ABCG2, URAT1/GLUT9, NLRP3, barrier, microbiome, and metabolome changes	Sample size, randomization, and blinding not stated in the review	Extends oral PDNV evidence to hyperuricemia-associated renal injury	Cannot separate urate lowering, intestinal disposal, and direct renal effects

Biodistribution refers to tissue-associated vesicle or tracer signals and does not by itself prove intact-vesicle delivery. Mechanistic validation distinguishes parallel biomarker changes from perturbation experiments testing functional requirement. “Not stated in the review” indicates that the corresponding methodological detail was not available in the submitted manuscript and should be checked against the original article before final submission.

Abbreviations: AKI, acute kidney injury; BUN, blood urea nitrogen; CLP, cecal ligation and puncture; ER, endoplasmic reticulum; GI, gastrointestinal; HELNV, honeysuckle-derived exosome-like nanovesicle; LPS, lipopolysaccharide; PDNV, plant-derived nanovesicle; SCr, serum creatinine; SCFA, short-chain fatty acid.

A 2026 study of *Tuber sinoaestivum*-derived vesicles reported protection against cisplatin-associated liver and kidney injury together with inflammatory, oxidative-stress, and microbiota changes (Yin et al., 2026). Because T. sinoaestivum is a truffle-forming fungus rather than a plant, it is retained only as comparative food-vesicle evidence. Synthetic carriers of plant bioactives, mammalian EVs, and computational studies without biological validation are likewise treated as adjacent rather than direct renal PDNV evidence.

## 4 Mechanisms of renal protection and strength of evidence

### 4.1 Oxidative stress and mitochondrial dysfunction

Renal tubular cells are highly dependent on mitochondrial energy production, and mitochondrial dysfunction contributes to cisplatin-, sepsis-, and ischemia-related kidney injury through ATP depletion, ROS generation, and secondary inflammatory and cell-death responses (Tang et al., 2020; Su et al., 2023). HELNVs improved mitochondrial structural and functional readouts in cisplatin AKI, while engineered Atractylodes vesicles restored mitochondrial membrane potential after LPS exposure (Gong et al., 2026; Yan et al., 2026). These findings support mitochondrial homeostasis as a relevant component of PDNV-associated renal protection.

Extra-renal studies of plant-derived vesicles from ginger, tea, and citrus, together with related food-derived vesicles such as those from shiitake mushroom, provide broader support for antioxidant and cytoprotective activity (Zhang et al., 2016a; Raimondo et al., 2015; Yang et al., 2020; Liu et al., 2020; Zu et al., 2021; Zhang et al., 2016b; Kilasoniya et al., 2023; Cai et al., 2022). They do not establish a common renal mechanism. In the kidney studies, mitochondrial improvement has generally been measured alongside changes in ROS, inflammation, and cell death without perturbing a defined vesicular cargo or upstream mitochondrial target, so the direction of causality remains incompletely resolved.

### 4.2 Inflammatory and immune responses

Inflammation contributes to AKI through tubular danger signals, endothelial activation, complement, and recruitment of innate immune cells (Sato and Yanagita, 2018; Tang et al., 2019). HELNVs reduced proinflammatory cytokines and increased IL-10, while AMEVLP and AMEVLP@miR altered macrophage-associated markers and NF-κB-related inflammatory readouts (Gong et al., 2026; Yan et al., 2026). Oral SMEV, EENV, and PLEN studies also reported lower renal or systemic inflammatory indices in parallel with changes in the intestinal environment (Liu et al., 2026; Yang L. et al., 2026; Yang Z. et al., 2026).

These observations do not imply that all PDNVs act through the same anti-inflammatory pathway. In orally administered systems, reduced renal inflammation may arise indirectly through barrier repair, microbial products, or circulating metabolites. Macrophage M1/M2 markers are also insufficient to define immune mechanism on their own, particularly in sepsis, where suppression of excessive inflammation must be considered alongside antimicrobial defense.

### 4.3 Regulated cell death and organelle stress

Regulated cell death is a recurrent downstream feature of kidney injury, but direct pathway evidence is uneven. The dandelion study provides the clearest example: SLC7A11 interference weakened vesicle-mediated protection, supporting a functional role for ferroptosis regulation (Zhang et al., 2026). By contrast, reductions in apoptosis in the HELNV and AMEVLP studies are consistent with protection but do not identify apoptosis as the primary vesicle-specific mechanism. Ferroptosis may also influence maladaptive repair after AKI, although long-term effects of PDNV treatment on AKI-to-CKD transition have not been established (Linkermann et al., 2014; Stockwell et al., 2017; Tang et al., 2021; Dixon et al., 2012; Guo et al., 2023).

LEVNs reduced endoplasmic reticulum stress together with tubular injury, crystal adhesion, and stone progression (Zhang et al., 2023). Calcium oxalate crystals can activate the unfolded protein response through disturbances in protein folding and calcium homeostasis (Cybulsky, 2017; Inagi, 2009), making ER stress biologically plausible in this setting. The current study links ER-stress attenuation to the anti-stone phenotype, but the responsible vesicular component and the relative contribution of specific UPR branches remain undefined.

### 4.4 Antifibrotic effects: evidence remains preliminary

TGF-β/SMAD signaling, fibroblast activation, and extracellular matrix deposition are central to renal fibrosis (Liu, 2011; Humphreys, 2018; Duffield, 2014; Meng et al., 2016; Meng et al., 2010; Liu, 2010). Reduced fibrotic markers have been reported in the SMEV diabetic-kidney-disease study and in related extra-renal vesicle or phytochemical systems, but the renal PDNV literature has not yet demonstrated reversal of established fibrosis. This distinction matters because lower lower TGF-β or α-SMA during early injury may reflect reduced upstream damage rather than a direct antifibrotic effect. A convincing antifibrotic claim would require treatment after fibrosis is established, together with quantitative matrix measurements and sustained functional outcomes.

### 4.5 Gut-kidney interactions

The gut-kidney axis is most relevant to orally administered PDNVs. Changes in barrier integrity, microbiota, microbial products, and circulating metabolites can influence systemic inflammation and renal injury, particularly in CKD and metabolic disease (Vaziri et al., 2013; Ramezani and Raj, 2014; Meijers et al., 2019; Evenepoel et al., 2017.; Yang et al., 2018; Mu et al., 2014; Wang et al., 2025). SMEV, EENV, and PLEN studies each reported renal improvement alongside intestinal or microbial changes, and AMEVLP@miR also altered the microbiota (Yan et al., 2026; Liu et al., 2026; Yang L. et al., 2026; Yang Z. et al., 2026).

At present, these relationships are better described as linked responses than as established causal pathways. Microbiota depletion, fecal microbiota transplantation, candidate-metabolite replacement, and route comparisons can determine whether the intestinal compartment is required for renal protection. Such experiments are especially important when little intact vesicle is expected to reach the systemic circulation after oral administration.

### 4.6 Metabolic regulation

Metabolic changes are prominent in the recent oral PDNV studies. SMEVs altered host metabolomic profiles in diabetic kidney disease, EENVs were associated with short-chain fatty acids and lipid metabolism in sepsis-associated AKI, and PLENs affected urate-related transport and metabolic readouts in hyperuricemia-associated injury (Liu et al., 2026; Yang L. et al., 2026; Yang Z. et al., 2026). These findings broaden the possible site of action beyond the kidney itself.

Metabolomic breadth, however, does not identify the metabolite that mediates protection. Absolute quantification, stable-isotope tracing, transporter inhibition, receptor perturbation, or replacement of candidate metabolites is needed to move from association to mechanism. In hyperuricemia, urate production, intestinal excretion, renal excretion, and urate-independent anti-inflammatory effects should be considered separately (Xiao et al., 2025).

### 4.7 Interpreting multicomponent effects

PDNV preparations can contain lipids, proteins, RNAs, and plant secondary metabolites, so simultaneous changes in several pathways are biologically plausible. They should not be interpreted as evidence that each altered pathway is an independent mechanism. The most informative experiments are those that identify a necessary vesicular component or upstream biological node and show loss of protection after its removal or perturbation. Dose and treatment timing also matter: pretreatment, early rescue, and delayed therapy address different biological questions and should not be pooled under a single efficacy claim.

## 5 Administration, biodistribution, and engineering of PDNV-based systems

### 5.1 Route of administration should reflect the proposed site of action

Oral administration is appropriate when the intended activity is primarily intestinal or microbiota-mediated and is also attractive for repeated treatment. The Atractylodes study shows, however, that oral delivery can be used for an engineered product proposed to have both intestinal and renal effects (Yan et al., 2026). Intraperitoneal or intravenous administration increases systemic exposure and has been used in the honeysuckle and dandelion studies (Gong et al., 2026; Zhang et al., 2026). Route therefore provides context for exposure but does not define the mechanism.

Dose reporting should be comparable across routes. Particle number should be reported together with at least one mass-based measure, the amount of starting plant material, administration frequency, treatment duration, and recovery yield where possible. Protein concentration alone is a poor dose metric because it can vary with purity and co-isolated material.

### 5.2 Renal accumulation is not equivalent to kidney targeting

Kidney-associated fluorescence can reflect circulating tracer, degraded membrane components, free dye, passive retention, or uptake by renal cells; it does not by itself demonstrate intact-vesicle delivery. Renal exposure may arise through glomerular filtration of sufficiently small particles or components, uptake from peritubular capillaries, resident phagocytes, or injury-related changes in vascular permeability and retention (Wang et al., 2026; Feng et al., 2026). Evidence for a defined ligand-receptor targeting mechanism is not yet available for the renal PDNV systems reviewed here.

Biodistribution studies should therefore combine fluorescent imaging with orthogonal approaches capable of following intact vesicles or a defined vesicular component. Radiolabeling, stable isotopes, membrane-specific tags, or cargo-specific assays can be coupled with time-resolved measurements in plasma, urine, kidney, liver, spleen, and lung. Cell-specific delivery requires additional evidence beyond whole-organ signal or anatomical colocalization.

### 5.3 Native PDNVs, engineered PDNVs, and reconstructed plant-lipid nanoparticles

Engineering changes both the function and, potentially, the identity of a plant-derived vesicle. Passive incubation, electroporation, sonication, extrusion, freeze-thawing, membrane fusion, and surface conjugation have all been used to introduce cargo or alter membrane properties in vesicle-based systems (Zhang et al., 2016b; Herrmann et al., 2021; Elsharkasy et al., 2020; Wiklander et al., 2019; Murphy et al., 2019; Huang et al., 2023). AMEVLP@miR provides direct renal proof of concept for loading an exogenous miRNA into a plant-derived vesicular chassis (Yan et al., 2026). For such products, loading efficiency alone is insufficient; encapsulation, protection from degradation, release, and cargo-specific activity in recipient cells need to be demonstrated.

A useful nomenclatural boundary is whether the original vesicular membrane architecture is retained. Surface-modified or cargo-loaded PDNVs can still be described as engineered PDNVs when the native membrane remains substantially intact. In contrast, plant lipids that are extracted, extensively disrupted, and reassembled into new nanoparticles are better described as reconstructed plant-lipid nanocarriers. Mammalian EVs modified with phytochemicals or hybrid inorganic components provide engineering precedents but remain adjacent evidence because the membrane chassis is not plant derived (Chen et al., 2026).

### 5.4 Oral formulation and gastrointestinal fate

Oral PDNVs encounter gastric acid, bile salts, digestive enzymes, mucus, epithelial barriers, food components, and resident microbes. Stability should therefore be assessed as a sequence rather than inferred from brief incubation at a single pH. Relevant measurements include particle size, membrane integrity, cargo retention, and biological activity after simulated gastric and intestinal exposure.

The formulation requirement depends on the proposed site of action. A product intended to act on the intestinal mucosa or microbiota may not require substantial systemic absorption, whereas a claim of direct renal delivery requires evidence that intact vesicles or an active cargo crosses the intestinal barrier and reaches the kidney. Enteric coatings, microgels, lyophilization, solutions, and capsules may each be useful, but they can also alter release, aggregation, and dose recovery.

Disease state can further change gastrointestinal behavior. CKD and diabetes alter intestinal physiology, and commonly used drugs such as antibiotics, proton-pump inhibitors, phosphate binders, and glucose-lowering agents can modify microbiota and luminal conditions. Oral pharmacokinetic and pharmacodynamic studies therefore need to distinguish local intestinal activity from systemic vesicle or cargo exposure.

## 6 Translational barriers and current clinical experience

### 6.1 Product identity and manufacturing consistency

Botanical source is part of the product definition. Plant species and variety, tissue, geographic origin, harvest conditions, maturity, storage, and post-harvest processing can alter vesicle yield and cargo. Medicinal plants add further variability from drying and other processing steps. Traceable source records should therefore be linked to particle yield, composition, and a small set of source-specific chemical or molecular markers.

Manufacturing also needs to move beyond small-batch ultracentrifugation. Closed filtration, size-exclusion, or density-based workflows are more compatible with scale-up, but each process can change the recovered particle population. Critical quality attributes should include particle concentration and size, morphology or membrane integrity, protein and lipid content, defined source-associated cargoes, microbial and endotoxin limits, residual pesticides, storage stability, and a potency assay related to the proposed mechanism (Pinedo et al., 2021; Thieron et al., 2024; Shokooh and Fereidoni, 2025; Viršilė et al., 2022; Xiao et al., 2018; Gimona et al., 2017). Independent batches should meet the same specifications before efficacy is interpreted as a product-level property.

### 6.2 Dose normalization and potency

Current studies report dose as protein mass, particle number, source-material mass, or administration volume, which limits comparison across preparations. At minimum, particle number should be paired with a mass-based measure and the amount of starting plant material and recovery should be reported when available. Potency assays should be linked to the proposed mode of action rather than to a generic PDNV marker. Depending on the product, this could involve a defined cargo, suppression of cisplatin-induced ROS, SLC7A11-dependent ferroptosis control, barrier function, or crystal-adhesion responses (Nguyen et al., 2020; Takakura et al., 2024).

### 6.3 Biodistribution and safety

Quantitative *in vivo* fate remains a major gap. Fluorescent signals are useful for screening distribution but cannot distinguish intact vesicles from free dye, exchanged lipids, or degradation products. Translation will require pharmacokinetic and tissue-distribution measurements that follow intact particles or defined vesicular components and relate exposure to dose and biological activity.

Plant origin does not establish safety. PDNV preparations may contain allergens, toxic plant constituents, pesticide residues, microbial products, RNAs, or metabolites that become relevant when the material is concentrated, engineered, or administered parenterally. Repeated-dose studies should address immunogenicity, complement activation, hemocompatibility, tissue accumulation, reproductive and genotoxic risk, and off-target effects of engineered cargoes. In cancer settings, renoprotection should also be shown not to compromise antitumor treatment.

### 6.4 What the existing clinical registrations actually investigated

No registered clinical trial identified in this review had evaluated PDNVs primarily for AKI, CKD, diabetic kidney disease, renal fibrosis, hyperuricemia-associated kidney injury, or nephrolithiasis as of 6 August 2026. Three nonrenal registrations illustrate different aspects of human plant-vesicle development. NCT01668849 evaluated an oral grape-derived preparation described in the registry in relation to chemoradiotherapy-associated oral mucositis and was completed with 60 participants, with no posted results. NCT04879810 tested oral ginger-derived exosome preparations, curcumin, or their combination in inflammatory bowel disease; only four participants were enrolled *versus* 90 planned, and no results were posted. NCT01294072 was designed to compare curcumin alone with plant-exosome-assisted curcumin delivery to normal and malignant colon tissue; its status was listed as unknown, with planned enrollment of 35 and no posted results (clinicaltrials.gov NCT01294072, 2011; clinicaltrials.gov NCT01668849, 2012; clinicaltrials.gov NCT04879810, 2018). The key characteristics and translational relevance of these human studies are summarized in Table 4.

**TABLE 4:** Representative human studies involving plant-derived vesicle preparations and their relevance to PDNV development.

Clinical trial	Population and intervention	Main question	Registry status and enrollment*	Relevance to PDNV development	Why it is not renal PDNV evidence
NCT01668849	Patients with head and neck cancer receiving chemoradiation; oral grape-derived preparation described in the registry in relation to grape exosomes	Whether the preparation could reduce oral mucositis; mucosal and immune outcomes were also assessed	Completed; n = 60; no posted results	Human experience with repeated oral exposure to an edible plant-derived vesicle-associated preparation and local mucosal outcomes	No kidney-disease indication; renal exposure, pharmacokinetics, renal safety, and renal efficacy were not evaluated
NCT04879810	Patients with inflammatory bowel disease; planned oral groups included ginger exosomes, curcumin, and ginger exosomes plus curcumin for 28 days	Safety/tolerability and changes in intestinal inflammation and symptoms	Completed; 4 enrolled *versus* 90 planned; no posted results	Relevant to oral intestinal administration and to separating intrinsic vesicle effects from vesicle-phytochemical combinations	Very small enrollment, no posted results, and no renal outcomes or kidney biodistribution measurements
NCT01294072	Patients undergoing surgery for newly diagnosed colon cancer; curcumin alone, curcumin with plant exosomes, or no treatment	Whether plant exosomes increase curcumin delivery to normal and malignant colon tissue; tissue curcumin concentration was the primary outcome	Status listed as unknown; planned n = 35; no posted results	Human precedent for plant-vesicle-assisted delivery of an exogenous bioactive compound	Addresses local colonic cargo delivery rather than kidney disease and does not establish systemic or renal delivery

* ClinicalTrials.gov records were rechecked on 6 August 2026, as reported in the manuscript. The terms “plant exosomes,” “grape exosomes,” and “ginger exosomes” follow the terminology used in the respective trial registrations and do not by themselves establish extracellular-vesicle biogenesis according to current EV criteria.

Abbreviations: PDNV, plant-derived nanovesicle.

These studies are relevant for different reasons. The grape study addresses repeated human mucosal exposure, the ginger study concerns oral intestinal administration and local inflammatory outcomes, and the colon-cancer study addresses plant-vesicle-assisted delivery of an exogenous compound. None establishes renal efficacy, kidney targeting, renal pharmacokinetics, or safety in patients with kidney disease. Their relevance is therefore translational rather than renal.

The limited human experience also argues against extrapolating clinical readiness from dietary familiarity. Oral exposure to a food-derived preparation, systemic administration of a concentrated vesicle product, and delivery of an engineered cargo present different pharmacological and safety questions.

### 6.5 Regulatory and reproducibility considerations

The regulatory category of a PDNV product will depend on source, processing, route, engineering, and therapeutic claim. Product identity, manufacturing consistency, impurities, potency, biodistribution, and risk controls therefore need to be developed as one package rather than as separate late-stage requirements (Takakura et al., 2024; Lener et al., 2015). For medicinal-plant products, botanical identification and cultivation records should be linked to particle attributes and potency, consistent with principles used for complex botanical products (U.S. Food and Drug Administration (FDA), 2016).

Dietary history can inform source selection, but it does not establish the safety of an isolated, concentrated, injected, or engineered nanovesicle preparation. Likewise, Generally Recognized as Safe (GRAS) status applies to a specified substance under specified food-use conditions and should not be extended automatically to a therapeutic vesicle product (U.S. Food and Drug Administration (FDA), 2023).

Reproducibility is also sensitive to seemingly technical choices. Homogenization intensity, rotor type, filter pore size, storage, particle-counting settings, protein assays, and fluorescent labeling can all change the recovered preparation or its apparent behavior (Van Deun et al., 2017; Vestad et al., 2017; Gardiner et al., 2016; Visnovitz et al., 2019). Reporting these parameters, using multiple independent batches, and retaining representative material for later analysis will make cross-study interpretation more reliable.

A practical development sequence is therefore to define the preparation and its critical quality attributes, establish a mechanism-linked potency assay, quantify relevant *in vivo* exposure, verify the proposed site of action, and then test efficacy and safety in appropriately controlled disease models. These steps are summarized in Figure 2.

**FIGURE 2:**
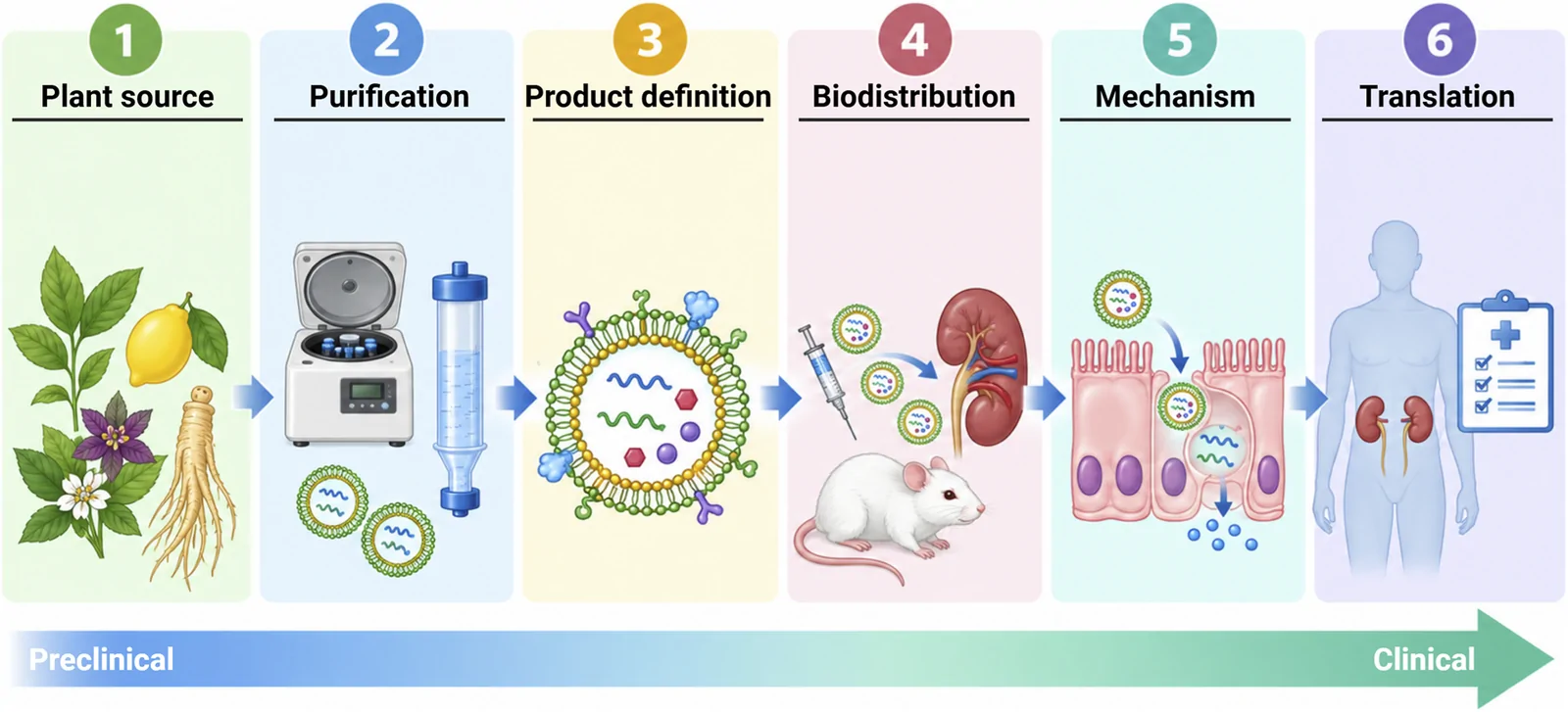
Key steps in the development of plant-derived nanovesicles for kidney-related applications. Development of a PDNV-based renal product begins with control of the botanical source, tissue origin, harvest conditions, and batch variability, followed by purification procedures that minimize co-isolated proteins, metabolites, membrane fragments, and other non-vesicular components. Product definition requires characterization of vesicle identity, composition, dose metrics, and biological potency, with native, engineered, and reconstructed plant-lipid preparations treated as distinct products. Biodistribution studies should determine whether kidney-associated signals represent intact vesicles, defined cargoes, or degradation products rather than relying on fluorescence alone. Mechanistic studies should move beyond parallel changes in signaling pathways to identify the vesicular components required for activity and test their causal contribution by depletion, perturbation, or rescue experiments. Translation then depends on reproducible manufacturing, mechanism-linked potency assays, appropriate dose selection, and repeated-dose safety evaluation before clinically interpretable renal studies can be considered. The lower arrow indicates the progression from preclinical product development toward clinical application; in practice, these steps are interdependent and may require iterative optimization.

### 6.6 Priorities before renal clinical translation

The immediate priorities are product definition, identification of active components, comparable dose and potency measures, quantitative biodistribution, and repeated-dose safety. Detailed disease-specific clinical endpoint schemes are premature while these basic features remain unresolved.

The next translational experiment should follow the proposed site of action. Products intended to act within the gut require evidence that intestinal or metabolic changes mediate renal benefit; products proposed to act directly in the kidney require evidence of renal exposure to intact vesicles or active cargo; engineered products additionally require loading, release, and cargo-specific activity.

Clinical indication should follow the mechanism that is best supported rather than the broadest possible disease scope. Early human studies would be most informative if they combine safety with measurable exposure or a short-term pharmacodynamic readout that can verify the proposed pathway. A negative mechanistic result at this stage would be more useful than a poorly interpretable efficacy signal.

Progression beyond early studies should require reproducible manufacturing and an exposure-safety relationship appropriate to the route of administration. This is particularly important for injectable preparations, repeated dosing, and products carrying exogenous nucleic acids.

## 7 Future research priorities

### 7.1 Identify the components that are necessary for activity

Most renal PDNV studies identify multiple changes in oxidative stress, inflammation, metabolism, or cell-death pathways without defining the vesicular component responsible for the effect. Omics analyses can nominate candidate lipids, proteins, RNAs, and metabolites, but necessity requires perturbation. Vesicle disruption, selective cargo depletion, nuclease or protease treatment, fractionation, and add-back or rescue experiments can distinguish activity of the intact vesicle from that of co-isolated soluble constituents. For engineered PDNVs, the introduced cargo should be evaluated separately from the intrinsic activity of the vesicle chassis.

### 7.2 Improve experimental comparability and causal inference

Current studies differ in plant source, purification, particle characterization, dose units, administration route, treatment timing, and outcome measures. Future reports should include randomization, blinding, sample-size justification where appropriate, prespecified exclusions, and independent production batches. Native PDNV studies benefit from matched plant extract, vesicle-depleted supernatant, and disrupted-vesicle controls, while engineered products additionally require unloaded-vesicle, free-cargo, and irrelevant-cargo controls.

Controls should also reflect the disease mechanism. Citrate-matched preparations are essential for lemon-derived vesicles, and cisplatin studies should assess antitumor compatibility. For gut-kidney claims, microbiota depletion or transplantation, metabolite replacement, transporter inhibition, or related perturbations are more informative than additional correlation networks. Similar logic applies to biodistribution: orthogonal tracking is needed when a mechanistic conclusion depends on renal delivery.

### 7.3 Resolve site of action and cell specificity

Whole-kidney signal is insufficient to identify the renal cell population that receives a PDNV or its cargo. Proximal tubular cells are a major target in cisplatin and ischemic AKI, whereas diabetic kidney disease involves podocytes, mesangial cells, endothelium, and tubules. Flow cytometry, spatial methods, single-cell approaches, kidney organoids, and microfluidic models can help distinguish cellular uptake from tissue-level accumulation (Wang et al., 2026; Feng et al., 2026).

Site of action is equally important for orally administered products. A renal benefit may follow local intestinal activity, microbial metabolism, systemic metabolites, direct vesicle or cargo exposure, or a combination of these routes. Experiments that separate these compartments are likely to be more informative than adding further downstream biomarkers.

Disease models should also match the question being asked. Preventive dosing, early rescue, and treatment of established injury should be analyzed separately, and chronic claims require follow-up long enough to distinguish transient biomarker improvement from durable renal protection.

### 7.4 Product-specific priorities and translational sequencing

The unresolved question differs by preparation. For HELNVs, active cargo and compatibility with cisplatin antitumor activity are central. For LEVNs, the vesicle effect needs to be separated from citrate and soluble lemon components. Dandelion vesicles require identification of the cargo responsible for SLC7A11 regulation and evaluation in the context of infection control. SMEVs, EENVs, and PLENs require causal tests of intestinal and metabolic mediation. AMEVLP@miR requires direct evidence of cargo encapsulation, release, and recipient-cell delivery.

Products that resolve these mechanistic questions can then be compared using common benchmarks for isolation, dose, stability, biodistribution, potency, and safety. Advancement to more complex pharmacokinetic studies, repeated-dose toxicology, and clinical testing should follow reproducible product definition rather than precede it.

This sequence keeps development anchored to the evidence available for each preparation and avoids treating all plant-derived nanovesicles as a single therapeutic class.

## 8 Conclusion

Plant-derived nanovesicles have shown preclinical activity in cisplatin- and sepsis-associated AKI, diabetic kidney disease, hyperuricemia-associated renal injury, and calcium oxalate nephrolithiasis. The current studies point to three overlapping modes of use: renal cellular protection, modulation of intestinal and metabolic pathways, and engineered cargo delivery. These categories are useful for organizing the literature, but they do not imply a common mechanism or equivalent strength of evidence across plant sources.

The evidence remains limited and heterogeneous. Renal fluorescence does not establish intact-vesicle targeting, microbiota and metabolomic changes do not by themselves demonstrate a causal gut-kidney mechanism, and many reported signaling changes have not been linked to a defined vesicular component. Progress will depend on clearer product identity, identification of essential effectors, standardized dose and potency measurements, quantitative biodistribution, and reproducible safety. Addressing these questions will determine whether the current proof-of-concept findings can develop into well-defined PDNV-based interventions for kidney disease.
